# The Potential Use of Fibrin Sealants in Burn Wound Management: A Comprehensive Review of Experimental and Clinical Studies

**DOI:** 10.3390/ebj6020032

**Published:** 2025-06-05

**Authors:** Christina Nikolaou, Maximos Frountzas, Emmanouil I. Kapetanakis, Dimitrios Stefanoudakis, Nikolaos A. Papadopulos, Stylianos Kykalos, Dimitrios Schizas, Dimitrios Iliopoulos

**Affiliations:** 1 Laboratory of Experimental Surgery and Surgical Research “N.S. Christeas”, School of Medicine, National and Kapodistrian University of Athens, 10509 Athens, Greece; kristenni@med.uoa.gr (C.N.); diliop@med.uoa.gr (D.I.); 2 Department of Plastic & Reconstructive Surgery, Burn Center, General Hospital of Athens “G. Genimatas”, 11527 Athens, Greece; 3 First Propaedeutic Department of Surgery “Hippocration”, General Hospital, School of Medicine, National and Kapodistrian University of Athens, 10509 Athens, Greece; stefanoudak@med.uoa.gr; 4 Department of Thoracic Surgery “Attikon”, University Hospital, National and Kapodistrian University of Athens, 10509 Athens, Greece; 5 Department of Plastic Surgery, “Eugenidio” University Hospital, National and Kapodistrian University of Athens, 10509 Athens, Greece; nikolaos.papadopulos@mri.tum.des; 6 Second Department of Propaedeutic Surgery “Laikon”, General Hospital, School of Medicine, National and Kapodistrian University of Athens, 10509 Athens, Greece; kykalos@med.uoa.gr; 7 First Department of Surgery “Laikon”, General Hospital, School of Medicine, National and Kapodistrian University of Athens, 10509 Athens, Greece; dschizas@med.uoa.gr

**Keywords:** fibrin, glue, sealant, burn, treatment

## Abstract

Fibrin sealants have been implemented in the management of burn wounds. They can be used either in combination with skin grafts for full-thickness burns or alone for treating superficial and deep dermal burns. The aim of this review was to provide critical insights regarding the efficacy of fibrin sealants in enhancing wound healing, improving graft adherence, and reducing complications. Therefore, evidence from experimental models and clinical trials was synthesized, underscoring the transformative role of fibrin sealants in modern burn care. This comprehensive review includes recent evidence on the potential benefits of fibrin sealants in the management of superficial and deep dermal or full-thickness burn injuries. Clinical and experimental evidence underscores some benefits in utilizing fibrin sealants in the management of superficial and deep dermal burn injuries, or in combination with skin grafts in full-thickness burns. Furthermore, fibrin sealants diminish postoperative pain and facilitate quick recovery for daily activities; however, controversy regarding their cost still remains. This review concludes that fibrin sealants could serve as a safe and effective therapeutic option for burn wound management. The safety and efficacy of their utilization, along with their wide availability and easiness to use, could make them an alternative treatment choice when a specialized plastic surgery service is not available, or in the emergency setting across different healthcare systems.

## 1. Introduction

Burn injuries can be classified into superficial burns, which exclusively affect the epidermis, or deep dermal burns, depending on the involved layer of the dermis and full-thickness injuries, characterized by the involvement of both the epidermis and dermis layers with potential extension into the subcutaneous tissue [1]. It has been estimated that 8.4 million new burn injuries occur annually globally, with approximately 180,000 deaths due to severe burn injuries per year worldwide [2,3]. Notably, the in-hospital mortality for patients with extended burn injuries is another important issue, as it ranges from 10% to 12%, while the maximum reported incidence can reach 72% if the TBSA exceeds 50% [4,5,6].

Consequently, burn injuries represent a significant clinical challenge for healthcare systems globally. Substantial resource allocation is necessary due to the intricate requirements for wound healing, reconstruction, eventual rehabilitation, and the reintegration of patients into their daily activities [7,8]. Moreover, the financial implications of delivering direct medical care for burn patients are notably elevated, usually reaching hundreds of thousands of dollars owing to protracted hospitalizations, complications, and the elaborative care needed [9].

Effective burn wound management requires a specialized approach, which includes the timely removal of necrotic tissue depending on the deepness of burn. On the other hand, wound care with antimicrobial agents and foam dressings could be considered as options in more superficial burn injuries. Moreover, complex surgical management with debridement and skin grafts in the case of full-thickness burn injuries might be necessary. Nonetheless, specialized plastic surgeons who offer the optimal surgical management after burn injuries are frequently unavailable in the emergency setting of various healthcare facilities, particularly in developing countries. Furthermore, general and emergency surgeons typically lack specific training in optimal burn management [10]. Under these circumstances, a need for a broadly accessible, easy to implement, and efficacious treatment modalities for burn injuries has emerged.

Biological fibrin sealants, which simulate the later steps of the human clotting cascade, could offer an alternative solution for such injuries, because they can facilitate tissue repair, wound healing, and hemostasis. Interestingly, they could be widely available and easily administered even by medical professionals without specialized training in plastic surgery. The goal of this review is to present a thorough analysis regarding their application in burn care, including both clinical and experimental studies, emphasizing their associated mechanisms in burn treatment and proposing their wider implications in clinical practice.

## 2. Fibrin Sealants’ Mechanism of Action

Fibrin sealants, also known as fibrin glues, are biological hemostatic agents developed to mimic the body’s natural coagulation process and support tissue healing. Initially introduced in the 1970s, their clinical application has expanded significantly across surgical disciplines, including general surgery, cardiovascular procedures, and burn management [11]. Unlike synthetic adhesives or mechanical hemostats, fibrin sealants integrate seamlessly into the host’s tissue, making them biocompatible and absorbable. They are particularly beneficial in cases involving oozing bleeding from raw surfaces or delicate tissue planes where suturing is ineffective or impractical. Over the past two decades, advances in biopharmaceutical manufacturing have led to the development of commercially available, virus-inactivated, standardized formulations approved by major regulatory bodies such as the (FDA), European Medicines Agency (EMA), and National Medical Products Administration Food and Drug Administration (NMPA). Their favorable safety profile, combined with their multifunctional properties in bleeding control, sealing, and wound healing, make fibrin sealants a valuable adjunct in modern surgical practice, especially in patients at high risk of bleeding or delayed healing [12].

Fibrin sealants simulate the final stage of the human’s natural clotting cascade. They mainly consist of thrombin and fibrinogen. When factor XIII and calcium are present in small portions, thrombin transforms fibrinogen into a monomeric form of insoluble fibrin, which is the stable final form of the sealant [13]. The final products of fibrin sealant contain fibrinogen in a concentration 20–40 times greater than in body fluids. These high concentrations facilitate the formation and enhance the quality of the fibrin clot. They also influence the adhesive strength, formation speed, network structure, permeability, and fiber thickness of the clot. On the other hand, adjustment of these concentrations enables the tailoring of the fibrin glue for specific uses. The concentrations of fibrinogen [40–50 mg/mL], factor XIII [20–480 U/mL], thrombin [4–1000 U/mL], and aprotinin [0–10,000 U/mL] vary in fibrin sealant compositions depending on the manufacturer [2,13,14]. Nevertheless, regardless of the different ingredients’ concentration, the clear benefit of fibrin sealant utilization consists of hemostasis, tissue adhesion, and the promotion of healing. These features have offered several advantages in burn treatment, leading to a wide increase in their use [15].

Fibrin sealants are generally considered safe and well-tolerated when used in burn wound management, with a low incidence of adverse effects. Their biocompatibility and biodegradability contribute to a favorable safety profile, especially when compared to synthetic adhesives. Most commercially available products undergo rigorous viral inactivation processes, reducing the risk of pathogen transmission. However, as with any biologically derived product, there remains a theoretical risk of allergic reactions, particularly in individuals with hypersensitivity to bovine or human plasma proteins [16]. Localized inflammatory responses, such as erythema or mild swelling, may occasionally occur. Rare but documented complications include immunogenicity, granuloma formation, or interference with wound healing when applied in an excessive volume [17]. Additionally, inadvertent intravascular injection can pose a thromboembolic risk, although this is exceedingly uncommon in topical applications [18]. Overall, fibrin sealants offer a safe adjunct in burn wound care, but their use should follow strict application protocols to minimize complications and ensure optimal healing outcomes.

## 3. Fibrin Sealants and Burn Wound Management

The present comprehensive review was designed according to the SANRA (Scale for the Assessment of Narrative Review Articles) bundle [19]. Medline (1966–2025), Scopus (2004–2025), EMBASE (1980–2025), Clinicaltrials.gov (2008–2025), Google Scholar (2004–2025), and the Cochrane Central Register of Controlled Trials CENTRAL (1999–2025) were explored by two authors (C.N. and M.F.). The main search strategy was as follows: (“fibrin” OR “sealant”) AND (“burn” OR “wound” OR “graft”) OR “clinical” OR “experimental”. We applied no filters regarding the study type, language, or publication date.

### 3.1. Experimental Studies

**Figure 1:**
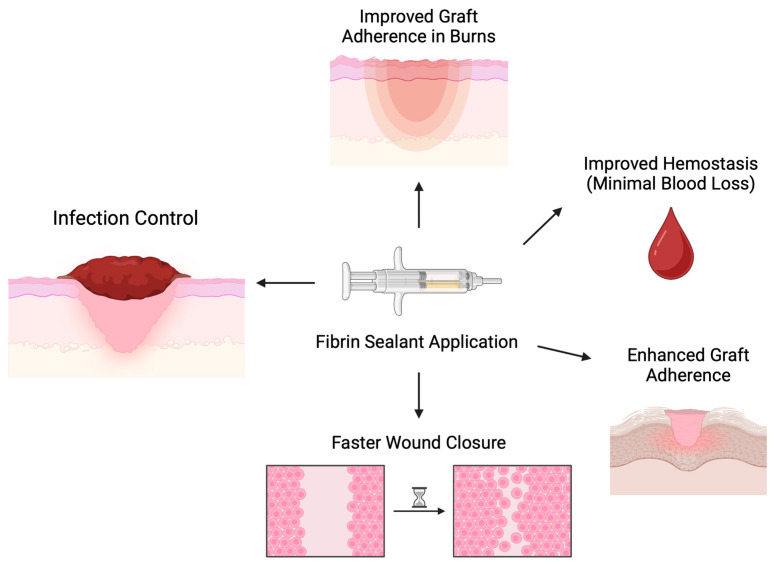
Mechanics and outcomes of fibrin sealant application in experimental skin grafting models. Fast wound closure was assessed by observation.

Various experimental models of skin graft utilization have demonstrated several advantages of fibrin sealants that could further contribute to burn wound management, as depicted in Figure 1. Firstly, Dyess et al. showed that the use of fibrin sealant enhanced the adherence of skin grafts and lowered the possibility of hematoma formation, thus resulting in improved graft survival rates and better wound healing [20]. The graft take rate was 87.6% ± 4.38%, with a median of 95%, whereas there was no slippage of the skin grafts, even without staples or sutures securing it. In addition, they observed a quick closure of the interstices, about 5.6 ± 0.5 days, with a median of 6 days. Finally, the amount of blood loss at the excision and graft sites was minimal, about 10 cc in total [20].

Likewise, Jabs et al. also investigated the effect of fibrin glue in skin grafts at infected sites. Using rat experimental models, they created wound beds using a dermatome, which was then inoculated with methicillin-sensitive, coagulase-positive *Staphylococcus aureus* [21]. Before proceeding to skin grafting, wound beds were treated with normal saline (Group I), fibrin glue with aprotinin (Group II), and fibrin glue alone (Group III). The comparison between Group I and Group II showed that Group II had a significantly higher percentage of skin graft success in highly infected wounds (80% vs. 35%; *p* < 0.005). Similarly, a higher skin graft success rate was observed when Group III was compared to Group I (85% vs. 35%, *p* < 0.003 in highly infected wounds) [21].

Furthermore, Balceniuk et al. investigated the effects of fibrin glue on the adhesive strength of skin grafts in areas with significant movement. The experiment was conducted in rats and compared the adhesive strength between skin grafts fixed with fibrin glue and those fixed with standard bolsters (control group) [22]. The strength was calculated on postoperative days (PODs) 1, 2, and 3 by measuring the force required to dislodge the graft from the recipient wound. There was a great difference between the experimental and control groups, with an average force of 719 g vs. 161 g on POD 1, 895 g vs. 257 g on POD 2, and 676 g vs. 267 g on POD 3 (*p* = 0.036, *p* = 0.029, and *p* = 0.024, respectively). Therefore, they concluded that the use of the fibrin sealants could offer a significantly greater adhesive strength to the graft during the critical phases of early healing, thus enhancing the chances of successful adherence [22].

An additional experiment conducted by Branski et al. investigated graft adherence after fibrin sealant application in a porcine full-thickness burn wound model [23]. After a full-thickness thermal contact burn, the injured area was excised, and after 24 h, it was covered by a skin graft that was fixed either with fibrin sealant in the form of slow-clotting spray or with standard skin staples. The clinical scores for graft adherence were significantly higher [*p* < 0.02] in the group of fibrin sealant compared to the control group on POD 2 and 5, whereas the scores for graft dislocation were significantly lower [*p* < 0.01]. However, there was no difference between the two groups regarding complications, such as wound contraction and hematoma, as well as epidermal and dermal thickness, 14 days postoperatively [23].

### 3.2. Clinical Studies

**Figure 2:**
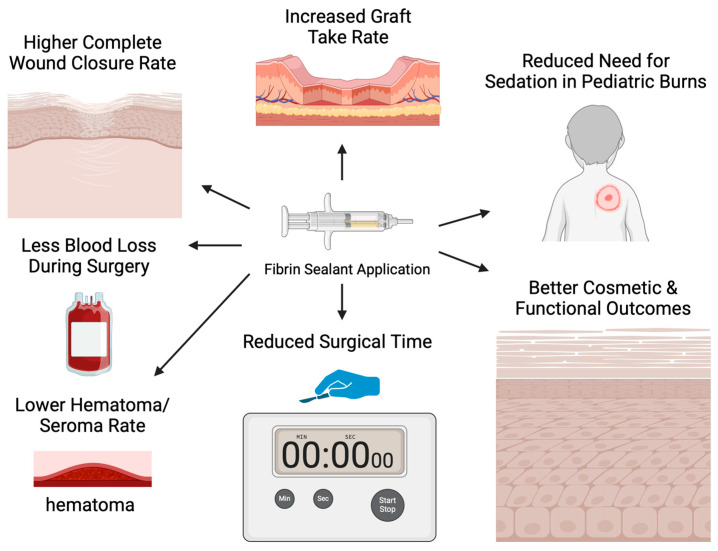
Outcomes of fibrin sealant application in the clinical setting.

Research on fibrin sealant applications with skin grafts has also been conducted in the clinical setting of burn injuries, corroborating the results of experimental studies, as depicted in Figure 2. Foster et al. investigated the effectiveness and safety of fibrin sealant utilization for securing autologous skin grafts in burn injuries during a phase 3 clinical trial, comparing fibrin sealant and suture fixation of skin grafts in 138 patients [18]. Wound closure was evaluated on POD 28 by three independent evaluators, using test site planimetry and photograph analysis, demonstrating 70.3% complete wound closure in the fibrin sealant group and 65.8% in the stapler group. The hematoma/seroma rate was significantly lower in the fibrin sealant (FS) group compared to the stapler (S) group (29.7%, 95% CI 22.2–38.1% vs. 62.3%, 95% CI 53.7–70.4%; *p* < 0.0001). Likewise, the rate of complete engraftment on POD 5 was higher in the FS group (62.3%, 95% CI 53.7–70.4%) compared to the S group (55.1%, 95%CI 46.4–63.5%). Ultimately, the FS group scored significantly better than the S group for all investigator-assessed outcomes, such as the quality of graft adherence (*p* < 0.0001), the preference for method of fixation (*p* < 0.0001), the satisfaction with graft fixation (*p* < 0.0001), and the overall quality of healing (*p* < 0.0001). The same pattern was reported for all patient-assessed outcomes, such as the anxiety about pain (*p* < 0.0001) and the treatment preference (*p* < 0.0001). The safety of fibrin sealant utilization was thus proved, as there were no serious related adverse events [24].

The effectiveness of fibrin glue on skin graft adherence, along with its safety, was also addressed in an observational prospective study conducted by Reddy et al. Sixteen patients who received split skin grafts were included and divided into two groups: in the first group, fibrin glue was applied to the recipient bed prior to grafting, while in the control group, the grafting was secured in the usual manner with a suture or with a skin stapler [25]. The results showed better graft adhesion with no hematoma or seroma formation in the fibrin glue group, compared to three cases with hematoma in the control group. On top of that, there was a significant reduction in surgical time in the fibrin glue group (17 ± 5 min vs. 20 ± 5 min, *p* < 0.05). Furthermore, the absence of any serious related adverse events, in conjunction with the great cosmetic outcomes, proved that fibrin glue is safe and effective for the attachment of skin grafts [25].

In addition, Han et al. conducted a study comparing fibrin glue with undiluted high-concentration thrombin (Group I) to sutures (Group II) for fixing split-thickness skin grafts. Their findings indicated that the fibrin sealant outperformed sutures and achieved a high graft take rate [26]. Specifically, after 5 days, the rates of hematoma/seroma formation and graft dislocation were 7.84 and 1.29% in Group I, and 9.55 and 1.45% in Group II, respectively. However, at 30-day follow-up, the rates of graft necrosis and graft take were 1.86 and 98.14% in Group I and 4.65 and 95.35% in Group II, respectively [26]. Another retrospective comparative cohort study by Muthukumar et al. examined the effectiveness of fibrin sealant compared to cyanoacrylate glue in securing grafts, and the resulting scar characteristics following skin grafting on areas affected by burns [27]. Outcomes from the 40 included patients showed a higher percentage of graft take at POD 15 for the fibrin sealant (Group I) compared to the cyanoacrylate glue (Group II) (95% vs. 90.2%, respectively). Regarding complications, two patients with graft failure (26.75%) were reported in Group I compared to five patients in Group II (38.16%). At the 6-month review, the subjective scorings, concerning pigmentation vascularity, thickness, and pliability were better in Group I. On top of that, histopathological and immunohistochemical staining revealed improved scar characteristics in Group I [27].

Hand burns represent a special clinical entity encompassing significant anatomical and functional challenges regarding their management. Increased mobility of this body area makes graft adhesion more difficult than in less mobilized body parts. Moreover, uneventful burn wound healing is more important than in other body parts, such as trunk or proximal extremities, because abnormal tissue scarring could lead to significant functional impairment [28]. Kim et al., in a prospective cohort study of 40 patients with hand burns, reported that using fibrin sealants with tourniquets led to less blood loss and improved graft success compared to treatment with epinephrine tumescence [29]. The estimated blood loss per cm was significantly lower (0.30 vs. 1.00 mL, *p* < 0.001), while the graft take rate was significantly higher in the fibrin sealant with tourniquet group (99.2% vs. 98.2%, *p* = 0.032) [29]. On the same basis, Boeckx et al. investigated the impact of fibrin glue in the treatment of dorsal hand burns regarding sensibility and mobility [30]. All included patients in this study were treated with full sheet skin grafts with or without fibrin glue. Based on follow-up evaluations, the group with fibrin glue reported better outcomes in two-point discrimination, with a mean of 1.01 cm vs. a mean of 1.39 cm in the group without fibrin glue. Similar results were reported for touch recognition, with a mean of 3.83 log force in the fibrin glue group vs. a mean of 4.41 log force on the dorsal surface of the hand, and 3.35 log force vs. 4.08 log force on the dorsal surface of the fingers, respectively. Finally, the fibrin glue group seemed to facilitate mobility as well. The most marked differences were reported in the flexion of the metacarpophalangeal (MCP) joints of all five fingers. Fairly good differences were also noted in the flexion of the proximal interphalangeal (PIP) joints [30].

Moreover, Vedung et al. demonstrated that applying fibrin sealants with skin grafts to infected burn wounds and difficult anatomical areas resulted in reduced inflammation, reduced fluid and blood accumulation, and thus improved healing [31]. Patients with previous failure of grafts due to bacterial infection, the movement of graft due to difficult anatomical areas, and bleeding were included in this study. Skin grafts were fixed with fibrin sealants in the form of glue or aerosol. Although the culture of all wounds revealed contamination, there was almost complete take of the skin grafts and healed residual areas with no additional intervention. Fibrin glue diminished the dead space under the skin graft, leaving no space for blood or serum accruement, thus reinforcing the fixation of the graft, leading to successful wound healing. There was only one out of twenty-two patients with a burn wound in the perineum, which was further infected due to diarrhea and healed after a second skin transplantation [31].

Furthermore, a significant study in children with burns examined the use of fibrin sealant for securing skin grafts and anesthetic needs [32]. This approach aimed to reduce the need for sedation in a pediatric cohort and demonstrated results similar to those achieved with skin staples, but requiring a lower use of sedations in the fibrin sealant group compared with the control group (1 versus 20, *p* < 0.0001) [32]. Finally, concerning the esthetic basis in a case report, a patient received a composite graft from the ear to repair the soft triangle of the nose. The use of fibrin glue in this instance promoted the successful integration of the graft and resulted in a pleasing appearance with minimal contraction of the graft [33].

On the other hand, meta-analyses and systematic reviews have examined the role of biological substances in burn treatment and confirmed those results. A systematic review and meta-analysis including 751 interventions by Grunzweig et al. concluded that fibrin glue could be as effective as staples for securing skin grafts, with fewer cases of hematoma and seroma, while they recommended its use for anatomically complicated areas [34]. After one week, there was complete adherence in 67.6% of grafts with fibrin sealant vs. 55.5% in those with staples (OR 1.45, *p* = 0.086). In addition, complete wound closure after one month was found in 80.2% with fibrin glue vs. 73.3% with staples (OR 1.34, *p* = 0.187). The rate of hematoma/seroma formation was lower in the fibrin group (38.2% vs. 64.7%, OR 0.487, *p* = 0.122), as well as the graft loss rate (12.6% vs. 1%, OR 0.891, *p* = 0.604) [34].

## 4. The Role of Fibrin Sealants in Postoperative Pain

Postoperative pain affects patients’ return to daily activities and their mental status, playing an important role in surgical decision-making regarding burn wound management. Boccara et al. concluded that pain scores were significantly lower with fibrin glue compared to staples for skin graft fixation (visual analog scale 1.66 vs. 4.33; *p* = 0.004) [35]. In addition, Foster et al. highlighted that pain scores were notably higher after staple removal following skin graft fixation. Patients reported a mean pain score of 6.2 ± 2.9 immediately after staple removal, which was significantly higher than the mean score of 3.3 ± 2.8 reported prior to the removal (*p* < 0.0001). Notably, 58.7% of patients needed extra pain relief or sedation during the staple removal procedure, suggesting that staples induced more pain, consequently concluding that patients experience less anxiety about pain when fibrin glue was used instead of staples (*p* < 0.0001) [24]. This has been implicated to be the most important mechanism through which fibrin sealants reduce pain following graft fixation. The avoidance of sutures or staple removal, which is provided by fibrin sealant utilization for graft fixation, ensures that healed burn tissue is not further handled after strong initial painkillers have been removed. However, future studies could investigate potential physiologic pathways regarding these observations, which could shed light from a molecular point of view.

Furthermore, a prospective randomized controlled trial by Healy et al. showed that patients treated with fibrin sealant and self-adhesive fabric dressing at their split skin graft donor sites reported significantly less pain (mean score 0.42 vs. 1.60; *p* < 0.001) and disability (mean score 0.48 vs. 1.71; *p* < 0.001) compared to those who only received self-adhesive fabric dressings [28]. This approach facilitates a faster return to regular activities [36]. A prospective study by Burton et al., which compared the postoperative pain between fibrin sealant and the staple fixation of grafts in extremities, showed promising results as well [37]. Patients with fibrin sealant experienced significantly less pain after surgery, with pain scores averaging 6.43 in the staples group and 4.86 in the fibrin glue group on POD 1 (*p* = 0.033). Similarly, the scores were 5.57 in the staples group and 3.29 in the fibrin glue group (*p* = 0.007) on POD 2. By POD 3, the average pain score was 4.00 for staples and 2.29 for fibrin glue (*p* = 0.011) [37]. Moreover, fibrin glue has shown adequate pain results when used in donor sites.

## 5. The Impact of Fibrin Sealants on Cost

On the other hand, despite the encouraging experimental and clinical outcomes presented above, current studies present controversial results regarding cost. A retrospective cost–benefit analysis comparing fibrin sealant and staples for fixing skin grafts in small burns indicates that fibrin sealants lead to fewer complications (<1% vs. 4%; *p* = 0.03) and faster patient discharge (mean 4.2 vs. 6.0 days; *p* < 0.001) for burns covering less than 10% of total body surface area (TBSA), resulting in cost savings (mean USD 4336 vs. USD 5082) [38]. Lower mean charges were also demonstrated in a retrospective study by Oltman et al., in which they compared fibrin sealant (FS) and the mechanical fixation (MF) of grafts [31]. The MF group had higher mean aggregate charges (USD 211,090) compared to the fibrin glue [FG] group (USD 149,907). On top of that, a significant reduction in the need for postoperative negative pressure wound treatment (FG: 16.7% vs. MF: 76.7%; *p* < 0.001), fewer 30-day postoperative visits (FG: 1.5 ± 0.78 visits vs. MF: 2.5 ± 0.03 visits; *p* < 0.001), and a shorter wound-adjusted operative time (FG: 51.8 min vs. MF: 67.5 min; *p* < 0.001) were observed [39].

Nevertheless, a similar retrospective review demonstrated no statistically significant differences between fibrin glue (FG) and sutures (S) for graft fixation in the overall direct costs (FG: USD 16,542 vs. S: USD 24,266; *p* = 0.545) or total charges (FG: USD 120,336 vs. S: USD 183,750; *p* = 0.496) [40]. However, this study highlighted the benefits of a reduced operative time and the complete removal from negative pressure wound therapy dressings (*p* = 0.612 and *p* < 0.0001, respectively) between the two groups [40].

## 6. Discussion

The present review shows that fibrin sealants could be safely used in combination with skin grafts, providing promising results compared to standard fixation. An experimental study has investigated the potential impact of fibrin sealants in tissue regeneration after burn injuries compared to conventional treatment with sulfadiazine [41]. After comparison with the conventional treatment (silver sulfadiazine cream) of dermal burn wounds and no treatment at all (control group), the fibrin sealant seemed to have more promising outcomes in terms of fibroblast and collagen deposition, as well as neovascularization. In particular, excessive [>80% of the wound area] fibroblasts were present in 50% of animals in the fibrin glue group compared to 30% of animals in the sulfadiazine group, and no animals in the control group (*p* = 0.002). The fibrin sealant group showed a higher rate of newly formed blood vessels, as 9 out of 10 animals (90%) had more than 10 new vessels. In contrast, only 1 out of 10 animals (10%) in the sulfadiazine group showed a similar level of vessel formation. On the other hand, no animals [0%] with more than 10 new vessels were reported in the control group (*p* = 0.000). The collagen levels were also significantly higher in the fibrin sealant group, which showed a dominant collagen concentration in 70% of animals, while only 10% in the sulfadiazine group showed such a collagen dominance. The control group had no animals (0%), with a dominant collagen concentration (*p* = 0.0001) [41].

Except from burn wound management, promising results regarding healing and regenerating capacity have been demonstrated in various applications of fibrin sealants in several surgical fields. Schwartz et al. evaluated the hemostatic properties of fibrin sealant by comparing the time of hemostasis, intraoperative blood loss, and rate of postoperative complications in liver resections [42]. Regarding time to hemostasis, fibrin glue had a tendency to lower the mean time (282 s) compared with standard agents (468 s, *p* = 0.06). On top of that, the percentage of patients with postoperative complications was lower in the fibrin sealant group (17.2% vs. 36.5%; *p* = 0.02). There were no statistically significant differences in the intra-operative blood loss between the two groups [42]. Furthermore, the hemostatic, adhesive, and regenerative qualities of fibrin sealant have been investigated in an experimental model of bowel perforation, showing encouraging results. Frountzas et al. assessed the effectiveness of a fibrin sealant compared to traditional suture techniques for treating confined bowel lesions in rats [43]. The results demonstrated less intra-peritoneal adhesions and lower hemorrhagic infiltration with thrombus formation (*p* = 0.042) in the fibrin glue animal group, diminished inflammatory reaction (*p* = 0.003), reduced fibrosis (*p* = 0.001), and greater tissue regeneration (*p* = 0.000) [43].

Existing evidence supports the safety of fibrin sealants’ utilization across various surgical fields, such as eye surgery, general surgery, and vascular treatments, without raising concerns [44,45,46,47]. Consequently, the safety of fibrin glue in the management of confined burn injuries, alone or in combination with skin grafts in extended burn injuries, is a crucial factor to consider. Such safety concerns seem to be unsupported considering the data provided by the aforementioned experimental and clinical trials. However, every clinician should be aware that only feasibility studies, which assess short-term outcomes and clinical parameters usually in a subjective way regarding the utilization of fibrin sealants in burn management and graft fixation, have been conducted so far. Implementation into clinical practice should carefully outweigh their potential benefits against possible risks in terms of cost, immunologic responses, and long-term outcomes. Therefore, large-scale prospective and even randomized multicenter trials will need to be conducted in order to confirm the safety and long-term effectiveness of fibrin sealants for the management of burn injuries across different clinical settings.

## 7. Conclusions

Fibrin sealants seem to promote skin grafting after burn injuries, showing promising results in clinical and experimental studies. Improved graft adherence for burn wound management, better infection control, improved hemostasis, and faster wound closure are some of the potential benefits of fibrin sealants that have been reported in experimental studies so far. Moreover, clinical feasibility studies have demonstrated higher complete wound closure rates, increased graft take rates, less intraoperative blood loss, lower hematoma formation, reduced surgical times, reduced need for sedation in pediatric burns, and better cosmetic as well as functional outcomes. Notably, there are experimental data implicating them as the first-line option for treating dermal burn injuries with promising results in terms of tissue regeneration compared to conventional sulfadiazine management. However, every clinician should be aware that the data published so far represent feasibility studies investigating safety and short-term outcomes. Therefore, the clinical benefits should be outweighed against the potential risks regarding cost and side effects. Future well-designed randomized controlled trials on larger samples would shed light on safety and long-term effectiveness in various clinical settings.

## Abbreviations

The following abbreviations are used in this manuscript:

TBSA	Total body surface area
FDA	Food and Drug Administration
EMA	European Medicines Agency
NMPA	National Medical Products Administration
POD	Postoperative day
FS	Fibrin sealant
FG	Fibrin glue
S	Suture
MF	Mechanical fixation
MCP	Metacarpophalangeal
PIP	Interphalangeal
